# A Mobile Health Intervention (LifeBuoy App) to Help Young People Manage Suicidal Thoughts: Protocol for a Mixed-Methods Randomized Controlled Trial

**DOI:** 10.2196/23655

**Published:** 2020-10-27

**Authors:** Jin Han, Lauren McGillivray, Quincy JJ Wong, Aliza Werner-Seidler, Iana Wong, Alison Calear, Helen Christensen, Michelle Torok

**Affiliations:** 1 Black Dog Institute University of New South Wales Sydney Australia; 2 School of Psychology Western Sydney University Sydney Australia; 3 Centre for Mental Health Research Australian National University Canberra Australia

**Keywords:** suicide prevention, mental health, eHealth, mHealth, mobile health, digital health, smartphone app, dialectical behavior therapy

## Abstract

**Background:**

Self-help smartphone apps offer a new opportunity to address youth suicide prevention by improving access to support and by providing potentially high fidelity and cost-effective treatment. However, there have been very few smartphone apps providing evidence-based support for suicide prevention in this population. To address this gap, we developed the LifeBuoy app, a self-help smartphone app informed by dialectical behavior therapy (DBT), to help young people manage suicidal thoughts in their daily life.

**Objective:**

This study describes the protocol for a randomized controlled trial to evaluate the efficacy of the LifeBuoy app for reducing suicidal thoughts and behaviors, depression, anxiety, and psychological distress, and improving general mental well-being in young adults aged 18 to 25 years.

**Methods:**

This is a randomized controlled trial recruiting 378 young adults aged between 18 and 25 years and comparing the LifeBuoy app with a matched attention control (a placebo app with the same display but no DBT components). The primary outcome is suicidal thoughts measured by the Suicidal Ideation Attributes Scale (SIDAS). The secondary outcomes are suicidal behavior, depression, anxiety, psychological distress, and general mental well-being. The changes in the levels of insomnia, rumination, suicide cognitions, distress tolerance, loneliness, and help seeking before and after using the app are evaluated in this study. The study also addresses risk factors and responses to the intervention. A series of items assessing COVID-19 experiences is included in the trial to capture the potential impact of the pandemic on this study. Assessments will occur on the following three occasions: baseline, postintervention, and follow-up at 3 months postintervention. A qualitative interview about user experience with the LifeBuoy app will take place within 4 weeks of the final assessment. Using linear mixed models, the primary analysis will compare the changes in suicidal thoughts in the intervention condition relative to the control condition. To minimize risks, participants will receive a call from the team clinical psychologist by clicking a help button in the app or responding to an automated email sent by the system when they are assessed with elevated suicide risks at the baseline, postintervention, and 3-month follow-up surveys.

**Results:**

The trial recruitment started in May 2020. Data collection is currently ongoing.

**Conclusions:**

This is the first trial examining the efficacy of a DBT-informed smartphone app delivered to community-living young adults reporting suicidal thoughts. This trial will extend knowledge about the efficacy and acceptability of app-based support for suicidal thoughts in young people.

**Trial Registration:**

Australian New Zealand Clinical Trials Registry ACTRN12619001671156; https://www.anzctr.org.au/Trial/Registration/TrialReview.aspx?id=378366.

**International Registered Report Identifier (IRRID):**

PRR1-10.2196/23655

## Introduction

Suicidal thoughts and behaviors are a global public health concern for adolescents and young adults owing to their life-threatening nature and high prevalence [1]. Globally, it is estimated that over 200,000 people aged between 10 and 29 years died by suicide in 2016 [2]. Suicide remains the second leading cause of death for this age group despite numerous prevention initiatives over the last decade [3]. Rates have increased across multiple countries and regions in recent years [4-6], and the increased rates are likely to be a lasting concern owing to the coronavirus pandemic and economic recession [7]. One possible approach to prevent youth suicide is to provide accessible and engaging interventions that can effectively reduce suicidal thoughts and behaviors in this age group.

Dialectical behavior therapy (DBT) is one therapeutic approach that has been shown to reduce suicide-related outcomes, including nonsuicidal self-injurious behavior and suicide attempts [8,9]. DBT combines principles from behaviorism, Zen, and dialectics [10] that aim to help clients improve their emotional and cognitive regulation to overcome problems, including intense mood change [11,12], impulsivity [13,14], and loneliness [15]. DBT was initially developed for persons diagnosed with borderline personality disorder [16], and there is increasing evidence to suggest that DBT is also effective in reducing suicidal thoughts, nonsuicidal self-injurious behavior, and suicide attempts in both adults [17] and adolescents [18,19].

Traditional face-to-face psychotherapies, including DBT, usually carry high economic costs and personal barriers that may prevent youth from accessing them [20]. Literature suggests that only 28% of adolescents and young adults with current, past year, or lifetime suicidal thoughts, plans, and/or attempts have accessed mental health services [21]. Potential barriers to mental health services include lack of time, preference for self-reliance, stigma, and service unavailability [21,22]. Digitally delivered interventions offer a new opportunity to improve access to support and to provide high fidelity and cost-effective treatment [23] at scale. Self-help smartphone apps that are designed to be used without professional guidance can help address these gaps by allowing people to seek help anonymously at a relatively low cost and at a time that suits them [24]. There is emerging evidence that adults readily access and benefit from suicide prevention interventions delivered via smartphone-based apps [25]. However, there have been very few empirical studies examining the efficacy of mental health apps for young people, and there are even fewer studies for apps that specifically target suicidal thoughts in this population. To our best knowledge, no studies have investigated the possibility of using a DBT-informed app to reduce suicidal thoughts in young adults.

To address this gap, we developed the LifeBuoy app, a self-help smartphone app designed to help young adults manage suicidal thoughts and negative feelings in daily life. It includes seven structured therapeutic sessions derived from DBT and incorporates the principles of positive psychology. The primary objective of the trial is to investigate the efficacy of the LifeBuoy app compared with a matched attention control condition in reducing suicidal thoughts in young adults at postintervention and a 3-month follow-up compared with baseline. The second objective is to assess the impact of the app on secondary outcomes, including suicidal behaviors, depression, anxiety, psychological distress, and general mental well-being. We are also interested in examining the changes in tertiary outcomes, including insomnia, rumination, suicide cognitions, distress tolerance, loneliness, and help-seeking intentions and behaviors at postintervention and a 3-month follow-up compared with baseline, and assessing the potential impacts of demographics, perseverance, negative events, such as COVID-19, and expectation of treatment success on participants. The final objective of this trial is to investigate adherence, satisfaction, and acceptability of the LifeBuoy app via survey questions, app usage data, and qualitative interviews.

## Methods

### Trial Design

**Figure 1:**
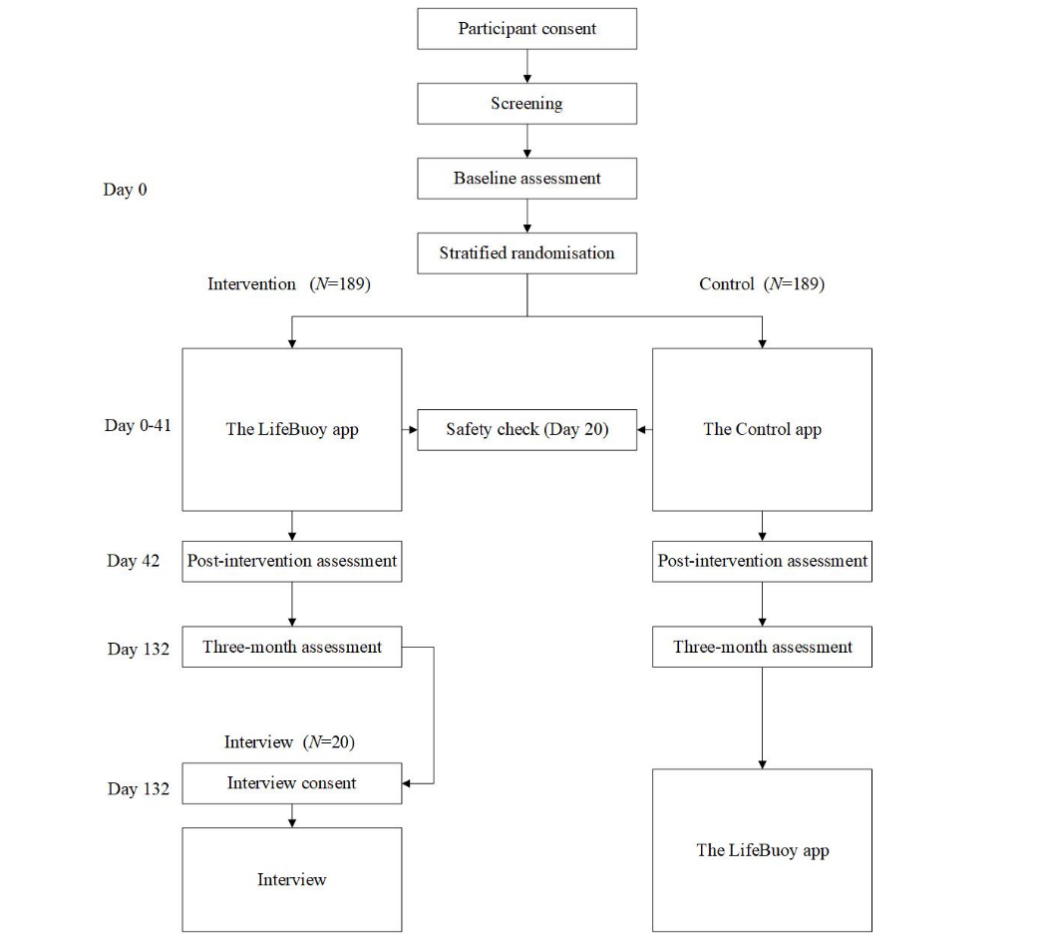
Study flow.

This study is a randomized controlled superiority trial with a matched attention control condition. Individuals are randomly allocated to either the intervention or attention control condition with a 2:2 allocation using a block design (four participants per block), stratified by gender and age group using an automated web-based platform tailored for this trial. Participants within each block (N=4) are randomly assigned to either of the two groups based on the six sequences, given a block size of four [26]. The trial has the following three measurement occasions: baseline, postintervention, and follow-up at 3 months postintervention. A qualitative interview about user experience with the LifeBuoy app will take place within 4 weeks of the final assessment (Figure 1).

### Blinding

In the trial, participants are not notified about whether they are assigned to an intervention or control group. Participants may still discern their condition assignment owing to the nature of the intervention content. All the investigators will be blinded to intervention assignment throughout the study period.

### Participants

Young adults aged between 18 and 25 years are eligible to participate in the trial if they (1) have experienced suicidal thoughts in the past 12 months, (2) have not made a suicide attempt in the past month, (3) do not have a diagnosis of psychosis or bipolar disorder, (4) currently live in Australia, and (5) are fluent in English. The inclusion criteria will be determined by an online self-report screening survey. They are not prohibited from receiving other treatments or interventions during the trial.

### Sample Size

Based on a systematic review and meta-analysis on online and mobile apps for suicidal thoughts and self-harm [27], an effect size of 0.30 (Cohen *d*) is expected between the intervention condition and the control condition at posttest for the primary outcome suicidal thoughts. Based on an attrition rate of 30% [28,29] and a 0.50 correlation between pre- and posttest scores of the primary outcome, a sample size of 189 for each condition (total N=378) will be required to detect the expected effect size with a power of 0.80 and alpha of .05. For the qualitative interviews, we will recruit a 10% subsample of individuals who participate in the intervention condition to assess their experience with the app (N=20), which is likely to reach data saturation according to prior studies [30,31].

### Recruitment

From May 2020, a community-based sample of participants is being recruited into the trial through targeted Facebook advertising hosted on the Black Dog Institute’s social media pages. The recruitment is still ongoing. A subgroup of the participants in the intervention group will be recruited for the qualitative interview via an expression of interest form provided before the final assessment.

### Intervention

The LifeBuoy app includes seven structured therapeutic sessions derived from DBT and incorporates the principles of positive psychology. It includes sessions on value identification, goal setting, psychoeducation, emotion regulation, and distress tolerance. These sessions are self-paced and delivered sequentially in the app. Each module takes approximately 3 to 7 minutes to complete. A mood tracker is included in the app to allow participants to rate their feelings multiple times per day. The app also contains a toolbox, which includes two distraction activities (popping bubbles and funny quiz) and additional tips for reducing distress (self-soothing tips and the Temperature, Intense exercise, Paced breathing, Paired muscle relaxation [TIPP] technique). Finally, there is a “help” button embedded in the app with direct links to Australian crisis lines. The LifeBuoy app was developed using a person-centered approach, involving young people with lived experience of suicidal thoughts, to understand and accommodate the perspectives of young people who will use the intervention. The design process of the app will be reported in a separate paper, which is currently in preparation.

### Matched Attention Control

Similar to the intervention app, the control app contains seven brief nontherapeutic education-based sessions. The topics are peripherally related to mental health and well-being, including confidence, performance stress, the importance of having goals, and the value of being present. Each session takes 2 to 3 minutes to read through. The control does not contain a mood tracker or toolbox but includes the “help” button for safety management. Participants in the control group will be granted access to the intervention app after they complete the 3-month follow-up assessment.

### Assessments

There are three data collection points as follows: baseline (day 0), postintervention (day 42), and 3-month postintervention follow-up (day 132) (Table 1). Data collection will be fully automated and online, with participants accessing the study website to register for the trial, download the app, and complete assessments.

**Table 1:** Summary of the primary, secondary, and tertiary outcome measures, risk factors, other measures, and data collection time points.

Outcomes measures		Scale	Baseline	Postintervention	Three-month follow-up
**Primary outcome**					
	Suicidal thoughts	Suicidal Ideation Attributes Scale (SIDAS)	Yes	Yes	Yes
**Secondary outcomes**					
	Suicide behaviors	Suicide attempt and self-harm	Yes	Yes	Yes
	Depression	Patient Health Questionnaire-9 (PHQ-9)	Yes	Yes	Yes
	Anxiety	Generalized Anxiety Disorder-7 (GAD-7)	Yes	Yes	Yes
	Psychological distress	Distress Questionnaire-5 (DQ-5)	Yes	Yes	Yes
	General mental well-being	Short Warwick-Edinburgh Mental Wellbeing Scale (SWEMWBS)	Yes	Yes	Yes
**Tertiary outcomes**					
	Insomnia	Insomnia Severity Index (ISI)	Yes	Yes	Yes
	Rumination	Repetitive Thinking Questionnaire (RTQ)	Yes	Yes	Yes
	Suicide cognitions	Suicide Cognitions Scale (SCS)	Yes	Yes	Yes
	Distress intolerance	Distress Tolerance Scale (DTS)	Yes	Yes	Yes
	Loneliness	Three-Item Loneliness Scale (TILS)	Yes	Yes	Yes
	Help-seeking intentions	General Help-Seeking Questionnaire (GHSQ)	Yes	Yes	Yes
	Help-seeking behaviors	Client Service Receipt Inventory (CSRI)	Yes	No	Yes
**Risk factors and other measures**					
	Demographics	Questions	Yes	No	No
	Perseverance	Short Grit Scale (SGS)	Yes	No	No
	COVID-19–related worry	Questions	Yes	No	No
	Negative events	Negative Life Events Scale for Students (NLESS)	Yes	No	No
	Expectations of treatment success	Questions	Yes	No	No
	Satisfaction with the app	Questions	No	Yes	No

### Outcomes

#### Primary Outcome Measure

##### Suicidal Ideation Attributes Scale

The primary outcome measure is the severity of suicidal thoughts assessed by the Suicidal Ideation Attributes Scale (SIDAS [32]). It consists of five questions pertaining to frequency of suicidal thoughts in the past month, controllability of suicidal thoughts, closeness to suicide attempt, level of distress associated with the thoughts, and impact on daily functioning. Each item is assessed on a 11-point scale (0-10). Item two (controllability) is reverse scored. Total scale scores on the SIDAS range from 0 to 50, with higher scores indicating more severe suicidal thoughts.

#### Secondary Outcome Measures

##### Suicide Behaviors

Participants’ previous suicide attempts and self-injury are assessed by eight questions developed for a previous suicide prevention trial [29]. Participants are invited to indicate whether they have attempted suicide in their lifetime and in the past 30 days on a three-point Likert scale, with responses of “No, never (0),” “Yes, once (1),” and “Yes, more than once (2).” They are also asked to report the number of suicide attempts over their lifetime and the number of months that they have been thinking about suicide. Apart from that, participants are asked to indicate whether they have experienced intentional self-injury in their lifetime on the aforementioned three-point Likert scale. If yes, they are asked to provide the number of times of their intentional self-injury and rate the severity of the worst injury in the past month on a three-point Likert scale, with responses of “No care was needed (1),” “Some care was needed (2),” and “Required medical care (3).”

##### Patient Health Questionnaire-9

The Patient Health Questionnaire-9 (PHQ-9 [33]) is a nine-item self-report questionnaire measuring the severity of depression. The scale assesses the frequency of occurrence of depression symptoms in the previous 2 weeks, with items rated on a four-point scale ranging from “Not at all (0)” to “Nearly every day (3).” Total scores on the PHQ-9 depression scale can range from 0 to 27, with higher scores reflecting more severe depression.

##### Generalized Anxiety Disorder-7

The Generalized Anxiety Disorder-7 (GAD-7 [34]) is a seven-item self-report measure designed to assess the severity of generalized anxiety symptoms over the previous 2-week period. Items are rated on a four-point scale, ranging from “Not at all sure (0)” to “Nearly every day (3).” Total scores on the GAD-7 can range from 0 to 21. Higher scores indicate higher levels of GAD symptoms.

##### Distress Questionnaire-5

The Distress Questionnaire-5 (DQ-5 [35]) is a five-item brief screening tool for identifying general psychological distress. Participants are asked to endorse the frequency of each item in the past 30 days on a five-point scale ranging from “Never (1)” to “Always (5).” Total scores range from 5 to 25, with higher scores indicating greater psychological distress.

##### Short Warwick-Edinburgh Mental Well-Being Scale

The Short Warwick-Edinburgh Mental Well-Being Scale (SWEMWBS [36,37]) is a shortened seven-item version of the 14-item Warwick-Edinburgh Mental Well-Being Scale (WEMWBS) [37], which was developed to assess mental well-being in the general population. It assesses mental well-being by asking about participants’ feelings and experiences over the previous 2 weeks. Responses range from “None of the time (1)” to “All of the time (5),” and raw item scores are summed and converted to a metric total score using the SWEMWBS conversion table [38]. Total scores can range from 7 to 35, with higher scores indicating higher levels of mental well-being.

#### Tertiary Outcome Measures

##### Insomnia Severity Index

The Insomnia Severity Index (ISI [39]) is a psychometrically sound, seven-item, self-report measure assessing the perceived severity of insomnia symptoms, the degree of satisfaction with sleep, interference with daytime functioning, noticeability of impairment, and concern caused by the sleep problems in the previous 2 weeks. Responses are reported on a five-point scale yielding total scores of 0 to 28. Higher scores indicate greater insomnia severity.

##### Repetitive Thinking Questionnaire

The Repetitive Thinking Questionnaire-10 (RTQ-10 [40]) is a transdiagnostic measure of engagement in repetitive negative thinking following distressing situations. The RTQ was developed to capture the underlying construct of recurrent negative thinking underlying mental health disorders such as depression and anxiety. Participants are requested to respond to the RTQ-10 on a five-point Likert scale, with responses ranging from “Not true at all (1)” to “Very true (5).” Total scores fall between 10 and 50, with higher scores indicating greater rumination.

##### Shortened Version of the Suicide Cognition Scale

The shortened version of the Suicide Cognition Scale (SCS [41]) is a self-report instrument consisting of nine items that are designed to measure suicide-specific cognition. The items contain statements consistent with the suicidal schemas of unbearability (eg, “I can’t cope with my problems any longer”), unlovability (eg, “I am completely unworthy of love”), and unsolvability (eg, “Nothing can help me solve my problems”). Items in the SCS are rated on a five-point Likert scale, with responses ranging from “Strongly disagree (1)” to “Strongly agree (5).” The instrument is scored by summing ratings across items, resulting in scores ranging from 9 to 45.

##### Distress Tolerance Scale

The Distress Tolerance Scale (DTS [42]) is a 15-item self-report measure designed to assess respondents’ perceived capacity to experience and endure negative emotional states. The DTS encompasses four subscales, including tolerance, appraisal, absorption, and regulation. Items are rated on a five-point Likert scale, with responses ranging from “Strongly disagree (1)” to “Strongly agree (5).” Higher mean scores indicate a greater tendency to withstand emotional distress.

##### Three-Item Loneliness Scale

The Three-Item Loneliness Scale (TILS [43]) is a brief self-report measure of loneliness. The three items that compose this scale were selected from the R-UCLA Loneliness Scale [44] and include the following: “How often do you feel that you lack companionship?” (relational connectedness); “How often do you feel left out?” (collective connectedness); and “How often do you feel isolated from others?” (general isolation). Response categories for the TILS are as follows: “Hardly ever (1),” “Some of the time (2),” and “Often (3).” Total scores are calculated by summing item scores, with higher scores indicating greater loneliness.

##### General Help-Seeking Questionnaire

The General Help-Seeking Questionnaire (GHSQ [45]) is used in the current trial to assess participants’ intentions to seek help for suicidal thoughts from a variety of sources. Respondents are invited to rate on a six-point scale, ranging from “Not applicable (0)” and “Extremely unlikely (1)” to “Extremely likely (5),” the likelihood of seeking help from three professional sources (school or university counsellor, mental health professional, and doctor/general practitioner), four informal sources (boyfriend/girlfriend, friends, parents, and other relative/family members), three telephone/online sources (phone helpline, internet website, and mobile app), or no one. An optional item “I would seek help from another source not listed above” is also provided. Higher scores represent stronger intentions to seek help.

##### Modified Client Service Receipt Inventory

The adapted version of the Client Service Receipt Inventory (CSRI [46]) is designed to collect information about use of health care and social care services over a retrospective period of the past 6 months. Respondents are asked to indicate whether they have used any services in the past 6 months owing to mental health problems, including suicidal thoughts, on a binary scale (“Yes”/“No”). Services include hospital services, mental health helplines, crisis support team, police/ambulance, contact with a range of mental health professionals (eg, social worker and counsellor), self-help groups, and other medically qualified doctors.

#### Risk Factors and Other Measures

##### Demographic Information and Baseline Variables

At the baseline assessment, participants are asked to provide their age, gender identity, gender assigned at birth, sexual orientation, contact information (email and mobile number), state and area they live in (ie, metropolitan or rural/remote), language spoken at home, who they live with at home, current relationship status, highest level of education completed, employment status, and whether they have ever experienced or been diagnosed with mental illness. Information related to their service use is also collected, such as whether they have ever seen a mental health professional for a mental health problem and used health or well-being apps.

##### Short Grit Scale

The Short Grit Scale (SGS [47]) is an eight-item measure assessing perseverance and passion for pursuing long-term goals. Half of the items are worded positively (eg, “I am diligent”), while the other half are worded negatively (eg, “New ideas and projects sometimes distract me from previous ones”) and are thus reverse scored. Items are rated on a five-point scale, with responses ranging from “Not like me at all (1)” to “Very much like me (5)” and the total scale score ranging from 8 to 40.

##### COVID-19–Related Worry

Ten items are used to assess the extent to which the coronavirus (COVID-19) pandemic influences participants’ perception of their symptoms (anxiety, depression, and suicidal ideation) and coping strategies. Items 1 to 3 relate to perceptions of symptoms and are rated on a five-point scale, with responses ranging from “Not at all (1)” to “All of the time (5)” (eg, “Do you think the COVID-19 pandemic has increased your anxiety levels more than usual?”). Items 4 to 6 relate to anticipated, experienced, and current worry and have been modified from a previous study of pandemic-related worry [48], with item 7 asking participants to indicate what aspects of COVID-19 are worrying them from a list of responses (eg, “not knowing when the pandemic will end”). Items 8 to 9 relate to the frequency and effectiveness of coping strategies used from a list of 14 equally balanced healthy (eg, “using social support” and “relaxation techniques”) and unhealthy (eg, “overeating/comfort food” and “avoidance/procrastination”) strategies. Participants are asked to rate their use of the strategies on a four-point scale (do not use, use less, use the same, and use more) and rate on a five-point scale whether they think each strategy used has been less effective during the pandemic, with responses ranging from “Not at all (1)” to “All of the time (5).” Item 10 asks participants to indicate if they have noticed any symptom improvement during or following the pandemic (none; yes, anxiety; yes, depression; yes, suicidal thoughts; and yes, other).

##### Modified Negative Life Events Scale for Students

The Negative Life Events Scale for Students (NLESS [49]) is designed to assess the experience of stressful life events among students. Respondents are asked to indicate whether they have experienced 25 negative life events (eg, death of a family member and being arrested) in the past year, and if yes, how stressful that event has been for them. In the adapted version of this measure, items are rated on a five-point scale, with responses ranging from “Not stressful (1)” to “Extremely stressful (5).” Higher mean scores indicate higher negative impact on life.

##### Expectation of Treatment Success

Four items were created to measure participants’ confidence and readiness in using an app to reduce suicidal thoughts, as well as the perceived importance of reducing suicidal thoughts and participating in research to reduce suicide risk. Items on this scale include “I am confident that people could reduce their suicidal thoughts using an app” and “I think that participating in a study that aims to reduce suicidal thoughts is an important thing to do.” These items are rated on a five-point scale, with responses ranging from “Strongly disagree (1)” to “Strongly agree (5).” Other items include “Please rate the importance of reducing your suicidal thoughts over the next 6 months,” with responses ranging from “Not important (0)” to “Very important (5),” and “Please rate your readiness to reduce your suicidal thoughts by using an app,” with responses ranging from “Not ready (1)” to “Completely ready (5).” Owing to the nature of these questions, this measure is administered only once during baseline assessment. Total scores indicate higher expectation of treatment success.

##### Satisfaction With the LifeBuoy App

Eighteen items were adapted from a previous study to assess participants’ satisfaction with the LifeBuoy app [50]. This measure consists of three parts. The first part contains seven statements related to the usability, readability, and helpfulness of the app, and the respondent’s intention to continue to use and recommend the app. Participants are asked to indicate whether they agree or disagree with each item. The second part of this measure comprises 10 questions pertaining to potential difficulties in using the app (eg, forgetting to use it and feeling worse after using it). Participants are asked to indicate whether they agree or disagree with each item. In the last part of this measure, they are asked to rate the overall helpfulness of the app on a five-point scale, with responses ranging from “Extremely unhelpful (1)” to “Extremely helpful (5).” Higher scores on each item indicate higher satisfaction with the app.

##### Treatment Adherence

Adherence to both the LifeBuoy app and control app is measured by the number of modules accessed and completed by participants, and the time spent on each module. The data are automatically collected via the app.

### Statistical Analysis

Mixed model repeated measures analyses with maximum likelihood estimation and an appropriate covariance structure will be used to evaluate the efficacy of the LifeBuoy app relative to the control condition. Within-person variation will be modelled by using an unstructured covariance matrix, and degrees of freedom will be estimated using Satterthwaite correction [51]. The primary outcome is the change in severity of suicidal thoughts across time (baseline to postintervention and postintervention to the 3-month follow-up). The mixed model approach incorporates all available data, including participants with missing follow-up data points, under the missing-at-random assumption, in accordance with the intention-to-treat principle. The same analytic approach will be used to examine temporal changes in secondary and tertiary outcomes, including depression, anxiety, psychological distress, general mental well-being, insomnia, rumination, suicide cognition, distress tolerance, loneliness, help-seeking intentions, and behaviors. The difference between the two groups on potential risk factors, including demographics, perseverance, COVID-19–related worry, negative events, and expectation of treatment success will be assessed by descriptive statistics.

In the interview, participants will be asked about their general feelings about the app and the design, their favorite and unfavorite features, the scenarios where they find the app useful, and other feedback about the app. The interview data will be analyzed using Braun & Clarke thematic analysis [52]. An inductive approach will be used to identify group themes. Two researchers will independently refine the themes and determine the final coding framework. Discrepancies will be resolved by a third researcher to ensure reliability of the process.

### Risk Management

In both the intervention and control versions of the LifeBuoy app, there is a help button that contains a list of 24-hour crisis support contacts that are publicly accessible nationwide. This button also contains a clinical psychologist contact button that allows participants to request a call from the team clinical psychologist.

Calls by the team clinical psychologist to participants will focus on ensuring the participants are safe and supported by their family and/or community if this is possible, and identifying how they can access the services they need. If participants express distress in relation to using the app or answering the assessment questionnaires, they will be reminded of their right to withdraw from the trial without penalty or explanation. In the event that a participant does not respond to the initial contact attempt, an email stating that contact was attempted and requesting for their availability will be sent. The psychologist will attempt to call the participants up to two times in the requested time. If the participant is still unavailable, another email stating that contact was attempted and containing referral sources will be sent.

At each survey timepoint (baseline, postintervention, and 3-month follow-up) and in the middle of the trial (3 weeks after commencing use of the LifeBuoy app), risk of suicide will be assessed using a standardized scale (SIDAS). If the SIDAS score is above 20 [32], indicating an elevated level of suicidal thoughts, an automated alert system will be triggered, in which a notification will be sent to the research inbox to notify the team clinical psychologist. An automated email will also be sent to the participants asking if they would like to receive a phone call from the team clinical psychologist as above. This email will also include a list of 24-hour crisis support contacts.

There are two levels of risk management in this project. The first is the trial steering committee, which consists of the research team, and the second is an independent Data Monitoring Safety Board (DSMB). The DSMB has three members who are experts in clinical trial conduct, statistics, and youth mental health. The trial manager will record the number of notifications at each assessment in a report for the DSMB and will notify the DSMB after each assessment period about how many alerts have been triggered and whether follow-ups have been carried out. Any serious adverse event will be reported to the DSMB. The DSMB will provide recommendations to the research team to continue the trial, temporarily pause the trial, or discontinue the trial owing to heightened risk or adverse events.

### Privacy, Confidentiality, and Data Management

The sensitive data that are collected in this study include participants’ responses to the surveys, usage data collected by the LifeBuoy app, and the audio records of the interviews.

The survey and usage data are collected by the Black Dog Institute eHealth research platform, a bespoke trial management system. Each participant will be assigned a unique identification code automatically at the time of registration on the Black Dog Institute research platform. When online survey data are exported for analysis, the research team will remove the identifiable information from the initial data set (ie, first name, mobile phone number, and email address will be removed). A deidentified extract of the data will be downloaded for analysis on a shared drive, which is password protected and approved by the university for storing highly sensitive data. The file used for analysis will only include the unique ID code and raw research data. Only named study personnel will have access to any identifiable information. For participant withdrawal, only named study personnel will access the register to identify the personal details of participants using their ID code.

Transcription of the interviews will be undertaken by a professional transcription service, and only deidentified audio will be provided to the agency. The agency will need to sign a confidentiality agreement before being provided with the recordings. Participants will be informed that deidentified audio will be provided to an agency for transcription service only. Upon completion of this project, all deidentified data will be stored in an archive on the server hard drive for a period of a minimum of 7 years, in accordance with University of New South Wales guidelines and the Australian Code for the Responsible Conduct of Research.

## Results

The current trial has ethical approval from the University of New South Wales Human Research Ethics Committee (HC190764). It has been registered in The Australian New Zealand Clinical Trials Registry (ACTRN12619001671156) and with the Therapeutic Goods Administration through the Clinical Trial Notification (CTN) scheme (CT-2020-CTN-00256-1-v1). Recruitment started on May 11, 2020. Data collection of the trial is expected to be complete by December 2020.

## Discussion

Preventing suicide in young people is a pressing global imperative [1]. The current mental health system addresses less than half of the need of support [21], leaving most young people to manage their symptoms alone. Self-help smartphone apps offer an opportunity to improve access to support for this population.

To our knowledge, the LifeBuoy study is the first trial to examine the efficacy of a DBT-informed smartphone app in reducing suicidal thoughts and related mental health symptoms in young adults. The app is innovative by integrating structured therapeutic sessions derived from DBT with distraction activities and mood assessments. If the LifeBuoy app is found to be effective, it may prove to be useful as a way to support young adults who do not usually seek help from mental health services or as an adjunct for those who do seek help. Because we have asked about current health service use, we will be able to determine for whom it is effective. We will also determine whether the app is useful for those in underdeveloped areas, where professional health sources are usually scarce. Through our qualitative interviews, we will find information on who our participants think it could be used.

There are few limitations we would like to acknowledge in the current trial design. First, the participants may not be blinded in terms of intervention allocation owing to the nature of the content of the app. All the researchers involved in this study will be blinded to the allocation at the time of analysis, thus maintaining the integrity of study results reporting. Second, the trial sample is being recruited from targeted Facebook advertising rather than by mail or phone. Samples recruited by Facebook have been found to present similar representativeness to convention methods in achieving age and gender distribution and are usually less costly [53]. Finally, the sample is being recruited during the COVID-19 period, which is likely to elevate participants’ levels of psychological distress and mental health symptoms [54]. We intend to examine this by measuring the relevant symptoms and the impact of COVID-19 in the surveys.

## Abbreviations

CSRI: Client Service Receipt Inventory
DBT: dialectical behavior therapy
DQ-5: Distress Questionnaire-5
DSMB: Data Monitoring Safety Board
DTS: Distress Tolerance Scale
GAD-7: Generalized Anxiety Disorder-7
GHSQ: General Help-Seeking Questionnaire
ISI: Insomnia Severity Index
NLESS: Negative Life Events Scale for Students
PHQ-9: Patient Health Questionnaire-9
RTQ-10: Repetitive Thinking Questionnaire-10
SCS: Suicide Cognitions Scale
SGS: Short Grit Scale
SIDAS: Suicidal Ideation Attributes Scale
SWEMWBS: Short Warwick-Edinburgh Mental Well-Being Scale
TILS: Three-Item Loneliness Scale
WEMWBS: Warwick-Edinburgh Mental Well-Being Scale
